# Clinicopathological Characteristics and Oncologic Outcomes of Endometrioid Ovarian Carcinoma: A Retrospective Study from a Tertiary Cancer Centre

**DOI:** 10.3390/biomedicines13102381

**Published:** 2025-09-28

**Authors:** Christina Pappa, Aakriti Aggarwal, Sally El Tawab, Sabina Nistor, Jennifer Thorne, Negin Sadeghi, Sanjiv Manek, Kezia Gaitskell, Sunanda Dhar, Jacopo Conforti, Federico Ferrari, Hooman Soleymani majd

**Affiliations:** 1 Department of Gynaecology Oncology, Churchill Hospital, Oxford University Hospitals, NHS Foundation Trust, Oxford OX3 9DU, UK; 2 Department of Obstetrics and Gynaecology, Jonh Radcliffe Hospital, Oxford University Hospitals, NHS Foundation Trust, Oxford OX3 9DU, UK; 3 Department of Obstetrics and Gynaecology, El Shatby Maternity University Hospital, Alexandria University, Alexandria 21526, Egypt; 4 Department of Cellular Pathology, Oxford University Hospitals, NHS Foundation Trust, Oxford OX3 9DU, UK; 5 Nuffield Division of Clinical Laboratory Sciences, Radcliffe Department of Medicine, University of Oxford, Oxford OX3 9DU, UK; 6 Department of Clinical and Experimental Sciences, University of Brescia, 25136 Brescia, Italy; 7 Nuffield Department of Women’s and Reproductive Health, University of Oxford, Oxford OX3 9DU, UK

**Keywords:** endometrioid ovarian carcinoma, synchronous endometrioid carcinoma, endometriosis, ovarian neoplasms

## Abstract

**Background/Objectives**: To evaluate the clinicopathological features, treatment, and survival outcomes and to identify independent prognosticators for recurrence and mortality in patients with endometrioid ovarian cancer. **Methods**: The medical records of patients diagnosed with endometrioid ovarian carcinoma between January 2010 and December 2022 were reviewed retrospectively. Demographic and disease-related data were evaluated. Kaplan–Meier survival analysis using log rank test and Cox regression was performed. **Results**: Seventy-six patients were included in the study. The median age at diagnosis was 54 years (range 31–86). A total of 85.5% of the patients were diagnosed with early-stage disease and 88.1% of the tumours represented low-grade carcinomas. Synchronous endometrioid endometrial cancer was confirmed in 19.7% of the cases. All patients underwent surgical management and 65.8% received adjuvant chemotherapy. Median follow-up time was 67.5 months. The 5-year disease-free survival and overall survival were 92.1% and 93.4%, respectively. The risk of cancer-related death was higher in advanced stages (HR = 13.86; 95% CI 2.16–57.17; *p* < 0.001) and in the presence of residual disease (HR = 15.18; 95% CI 2.36–87.17; *p* < 0.002). Residual disease and advanced stages were also identified as independent risk factors for disease relapse with HR = 16.04 (95% CI 2.61–93.7; *p* < 0.002) and HR = 11.73 (95% CI 1.92–41.6; *p* < 0.001), respectively. **Conclusions**: Endometrioid ovarian carcinoma usually affects younger patients with the majority of the cases representing low-grade carcinomas diagnosed at early stages. Residual disease and advanced stages are independently associated with inferior survival outcomes. There was no significance of lymph node dissection and adjuvant chemotherapy in the overall and recurrence-free survival rates. Further research focusing on molecular profiling should aim to define the prevalence and the prognostic value of major molecular alterations and develop precise stratification models to plan personalised treatment for optimal care.

## 1. Introduction

Ovarian cancer (OC) represents the eighth most common cause of cancer-related deaths in women and the leading cause of gynaecological-related deaths worldwide [1]. Endometrioid ovarian carcinoma (EOvC) represents the second most common type of epithelial OC accounting for 10% of primary ovarian carcinomas [2,3]. It typically presents at an earlier stage compared to other ovarian cancer subtypes and it is therefore associated with favourable prognosis and higher 5- and 10-year overall survival rates [2,4].

Recent research supports that each type of OC is thought to have a different precursor lesion which can explain its biological behaviour and prognosis. Endometrioid OC and clear-cell adenocarcinomas have been linked to endometriosis and are widely described as endometriosis-associated ovarian carcinomas (EAOCs), frequently arising from ectopic endometrium in the ovary [5,6].

Several studies have shown that endometrioid ovarian cancer represents a distinct biological clinical, pathological, and molecular entity and should be regarded as such in the development of specific clinical approach and management [7,8,9].

Treatment for endometrioid ovarian cancer typically entails maximal cytoreductive effort aiming for complete macroscopic resection of the disease including peritoneal and retroperitoneal staging with cytology examination and pelvic and/or para-aortic lymph node assessment [10,11]. Since most of these cases are diagnosed at an early stage with a favourable prognosis, fertility-sparing surgery can be considered in selected cases, following meticulous counselling and multidisciplinary input [12,13].

Adjuvant treatment is currently considered depending on the stage, grade, and characteristics of the tumour, as adjuvant chemotherapy is associated with survival benefits in patients with inadequately staged and grade 2 stage I carcinomas [14,15].

Endometrioid ovarian cancer is subclassified by histologic grading similar to that used for endometrial carcinomas and it can be associated with synchronous endometrial endometrioid adenocarcinoma or endometrial hyperplasia in 15–30% of the cases [16,17]. Despite often clonally related, synchronous endometrioid ovarian and endometrial carcinomas typically present indolent clinical behaviour [18,19,20].

Tumour histotyping supported by specific immunohistochemical panels are used to classify the distinct categories of ovarian carcinomas [21]. The development and implementation of molecular analysis has further refined the different molecular subtypes with particular behaviour that signifies tailored management [5,22]. This has also led to more targeting treatments; however, there is a huge need to improve molecular characterisation in order to make possible the integration of novel adjuvant therapies (PARP-inhibitors; immunotherapies) [23].

The aim of the study is to assess the clinicopathological features, treatment outcomes, and survival patterns and to identify independent prognosticators for recurrence and mortality in patients with endometrioid carcinoma of the ovary managed in a single tertiary cancer referral centre.

## 2. Materials and Methods

We performed a retrospective analysis of patients diagnosed with endometrioid carcinoma of the ovary treated in Oxford University Hospitals, NHS Foundation Trust, a tertiary cancer referral centre (part of the Thames Valley Cancer Alliance Network serving a patient catchment area of 2.3 million), between January 2010 and December 2022.

The study was registered as a service evaluation project (registration number 7049) with no patient identifiable data. All the participants had signed an informed written consent for potential future research purposes at the time of primary treatment. The service evaluation protocol was registered in accordance with the Oxford University Hospitals Trust requirements. The design, analysis, interpretation of data, and drafting and revisions of the study were conducted in accordance with the Helsinki Declaration.

Patients with histological diagnosis of endometrioid ovarian carcinoma and no previous surgery concerning endometrial malignancy (primary surgery) were recruited. Exclusion criteria included patients with histology other than endometrioid ovarian carcinoma, patients with concomitant second primary malignancy other than low-grade endometrioid endometrial cancer, patients that received primary treatment elsewhere and presented at the time of recurrence, and patients with inadequate follow-up data.

We retrospectively reviewed data from electronic medical records of the eligible patients. All patients had imaging with computerised tomography (CT) scan radiological staging of the disease. Multidisciplinary team (MDT) meeting consensus was sought before the implementation of the treatment plan.

All eligible patients had surgical staging according to national guidelines. A re-staging procedure including peritoneal and/or retroperitoneal assessment by board-certified gynaecologic oncologist was performed in patients who did not undergo surgical staging at the time of diagnosis.

The extracted data included the age, menopausal status, body mass index (BMI), Ca125 status, and underlying comorbidities of the patient, length of hospitalisation, details about the surgical procedure such as route and type of surgery, and achievement of complete resection. Complete resection with no visible tumour was recorded as no residual disease (R0 resection), while minimal residual disease and gross residual disease were recorded when there was visible disease <1 cm and >1 cm, respectively. We recorded the final staging and tumour grading, the presence of endometriosis, and potential synchronous primary low-grade endometrioid endometrial carcinoma. Intraoperative, acute, and late post-operative complications as well as potential readmission were also recorded. For the patients who received adjuvant therapy, we recorded the type of therapy, the regiment, and the associated complications, whenever available.

Follow-up was recorded in months from the date of surgery up to the study period when the patients were last tracked. Follow-up was every 3 months for the first 2 years and then every 6 months up to completion of 5 years after completion of treatment, followed by annual appointments if required. Recurrence was recorded by histological confirmation of disease in tumour biopsy and/or the appearance of new lesions on imaging. Disease-free survival (DFS) was measured from the date of cytoreductive surgery until the date of first recurrence or death from any cause and overall survival (OS) from the diagnosis date until death.

Data analysis was performed using the IBM SPSS Statistics version 29.0 software. Continuous variables are presented as mean ± standard deviation or median (range), and categorical variables are presented using frequency (n) and percentage (%). Descriptive statistics were used to summarise the demographic and clinical characteristics of patients. We used the Kaplan–Meier method to perform disease-free survival (DFS) and overall survival (OS) analysis and log rank tests were used to compare survival rates. Potential risk factors for recurrence and mortality were assessed and analysed using univariate and multivariate Cox regression analysis. Hazard ratios (HR) were reported with their 95% confidence intervals (95% CIs). A *p*-value < 0.05 was considered statistically significant.

## 3. Results

A total of 93 patients were diagnosed with endometrioid ovarian cancer within the study period. Seventeen patients were excluded; three due to concomitant malignancy other than low-grade endometrioid endometrial cancer, two patients had mixed endometrioid and clear-cell ovarian pathology, five patients received primary treatment elsewhere, and seven patients were lost in follow-up.

Finally, 76 patients were included in our study. The demographic data, clinicopathological characteristics, treatment strategy, and outcomes are presented in Table 1. The mean age at diagnosis was 56.1 ± 12.9 years (range, 31–86 years), with 42.1% of the patients being below the age of 50 years old. Postmenopausal status was recorded in 52.6% of patients. The main route of surgery was laparotomy 72.4 (55/76) and 85.5% (65/76) of the patients underwent hysterectomy. One patient was pregnant at initial diagnosis and underwent completion surgery postnatally and three patients underwent completion surgery for peritoneal and lymph node staging. Many of the patients were diagnosed with stage I and stage II disease and only 11 patients (14.5%) were diagnosed with Stage III endometrioid ovarian carcinoma. Grade 1 and grade 2 tumours were confirmed in 88.1% of the cases. Seventy-two patients (94.7%) had no residual disease, four patients (5.3%) had residual lesions; three patients had residual lesion less than 1 cm and one patient less than 2 cm. Intraoperative tumour rupture occurred in eight cases resulting in stage IC1 disease necessitating adjuvant chemotherapy. Adjuvant chemotherapy was also offered in disease stages greater than 1C1, inadequately staged cases, and grade 2–3 tumours. Adjuvant treatment (carboplatin ± paclitaxel regime) was administered in 65.8% of the patients. Carboplatin monotherapy was used in nine patients; five due to frailty, three had severe pre-existing neuropathy, and one with underlying severe allergic reaction (Table 1).

**Figure 1:**
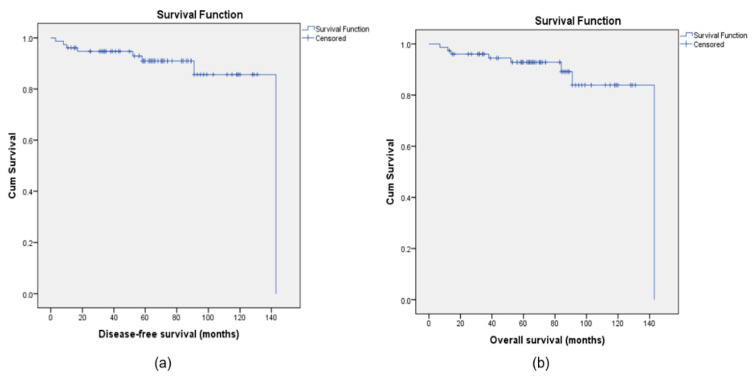
Kaplan–Meier curves for disease-free survival (DFS) (**a**) and overall survival (OS) (**b**) for the population study.

After a mean follow-up of 69.5 months (95% CI: 62.38–76.64), 5-year DFS and OS of the entire cohort were 92.1% and 93.4%, respectively (Figure 1).

The recurrence rate in our study was 17.1% (13 patients). More than half of the recurrences (61.53%) occurred within three years after surgery and 92.3% within five years. Only one patient (7.7% of recurrences) experienced recurrence of disease more than 5 years (77 months) after surgical management. Mean surgery to recurrence and recurrence to death intervals were 65.82 months (range 3–143) and 19.81 (range 4–67) months, respectively.

A total of 76.9% (10/13) of the recurrences presented with symptoms. Three cases were diagnosed due to raised Ca 125 followed by further investigation with imaging and not a single recurrence was diagnosed in routine follow-up clinical examination of asymptomatic patients. The most common symptoms of recurrence were pelvic pain (seven cases) and gastrointestinal symptoms (three cases). Two cases were also presented with rectal bleeding and two with renal impairment. The proportion of patients who have relapsed had received adjuvant chemotherapy is 76.9%. The relapsed cases are summarised in Table 2.

Univariate Cox regression analysis, performed for all variables presented in Table 1, has shown that the risk of disease relapse is related to the presence of residual disease (HR = 7.82; 95% CI 2.71–15.54; *p* = 0.003), advanced stages (HR = 5.37; 95% CI 1.98–14.72; *p* = 0.001), and positive Ca 125 levels (HR = 2.72; 95% CI 1.11–6.55; *p* = 0.025). The risk of cancer-related death was lower in the presence of endometriosis (HR = 0.12; 95% CI 0.14–0.96; *p* = 0.045) and higher in residual disease (HR = 8.01; 95% CI 1.94–16.96; *p* = 0.003), advanced stages (HR = 4.53; 95% CI 1.72–11.92; *p* = 0.002), grade 3 tumours (HR = 3.83; 95% CI 0.89–9.96; *p* = 0.004), positive Ca125 (HR = 2.60; 95% CI 1.09–6.19; *p* = 0.025), and in the presence of intraoperative complications (HR = 1.72; 95% CI 1.04–2.83; *p* = 0.035).

**Figure 2:**
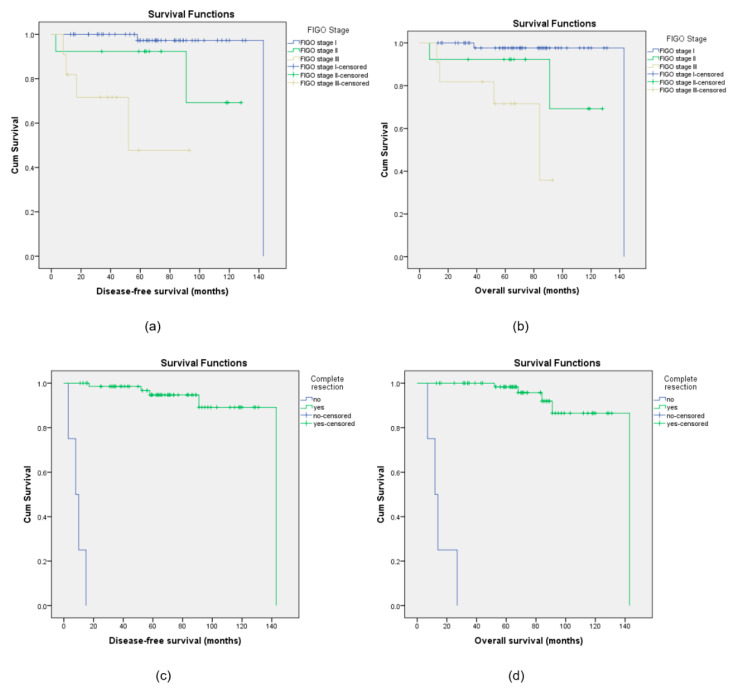
Kaplan–Meier curves for disease-free survival (DFS) and overall survival (OS) for the different stages of disease (**a**,**b**) and for complete or non-complete resection (**c**,**d**).

However, multivariate Cox regression analysis after the adjustment of the covariates confirmed that the stage of the disease and the presence of residual disease are the only independently related factors affecting the risk of recurrence and death from disease. The risk of cancer-related death was higher in advanced stages (HR = 13.86; 95% CI 2.16–57.17; *p* < 0.001) and in the presence of residual disease (HR = 15.18; 95% CI 2.36–87.17; *p* < 0.002). Residual disease and advanced stages also have a significantly higher risk of disease relapse with HR = 16.04 (95% CI 2.61–93.7; *p* < 0.002) and HR = 11.73 (95% CI 1.92–41.6; *p* < 0.001), respectively (Figure 2).

## 4. Discussion

This retrospective study aimed to assess the clinicopathological features, treatment, and survival outcomes and to identify independent prognosticators for recurrence and mortality in patients with endometrioid ovarian cancer treated in a single centre in a 12-year period.

The median age of diagnosis in our study cohort was 54 years, which is in line with similar studies and accounts for the earlier onset of endometrioid carcinoma compared with other types of ovarian cancer [14,24,25].

Atypical endometriosis has been widely described as a malignant precursor to endometrioid ovarian carcinomas with a significantly higher diagnosis rate of 20–45% in women with endometrioid ovarian cancer [26,27,28,29]. In this cohort, the presence of endometriosis has been histologically confirmed in 53.9% of the patients with endometrioid ovarian cancer. However, multivariate analysis has not proven that the presence of endometriosis represents an independent prognostic factor, which is in line with published articles [16,30]. Existing literature has shown that patients with endometriosis-associated ovarian cancer had favourable prognosis with higher survival rates [31]. This could be partially attributed to the earlier incidental detection of the disease due to the presence of endometriosis which usually initiates further investigation. The role of endometriosis in the development and prognosis in endometrioid ovarian carcinomas remains to be clarified [32].

In our study, diagnosis of synchronous endometrioid ovarian and endometrial carcinomas accounted for 19.7% of the cases, which is in accordance with the reported data in the literature [24,33]. Taking into consideration that up to 1/3 of the patients diagnosed with endometrioid ovarian carcinoma might have co-existing endometrial pathology, potential comprehensive evaluation of the endometrium might be considered for precise diagnosis and optimal management.

Several studies have shown high degree of concordance between the histologic and molecular subtypes of synchronous endometrial and ovarian tumours [34,35,36]. The Cancer Genome Atlas (TCGA) molecular classifiers for endometrial carcinoma has categorised ovarian endometrioid carcinoma into prognostically significant groups and several studies have indicated that ovarian endometroid carcinoma could be classified in clinically significant subgroups by testing for molecular surrogates likewise in endometrial cancer [37,38]. Endometrioid ovarian carcinomas carrying MMR and POLE alterations seem to represent a subgroup with favourable prognosis, while abnormal p53 indicates a worse prognosis [37,39]. A recent systematic review has also shown that the prognostic value of the TCGA groups was similar between endometrioid ovarian and endometrioid endometrial carcinomas, despite the differences in the frequency and pathological features of each group [40].

Endometrioid ovarian carcinomas show broad morphological similarities to their endometrial counterparts with the most common molecular alterations in endometrioid OC including mutations in CTNNB1 (31–53.3%), PIK3CA (15–40%), ARID1A (30%), and PPP2R1A (7–16.6%) [39,41,42]. Unfortunately, evaluation of molecular prognostic features in the study population was not feasible due to the limited data related to the molecular background of the tumours.

A recent retrospective multicentre study which aimed to uncover the distinct biological characteristics of endometrioid ovarian carcinoma, conducted a comprehensive molecular investigation, involving patients from three European centres, and has shown that endometrioid ovarian carcinoma demonstrates specific biological features with an IHC profile similar to that of endometrial endometrioid carcinoma, but with a gene expression profile more in keeping with ovarian clear-cell carcinoma [22]. Overall, endometrial ovarian carcinoma demonstrates substantial clinical and molecular heterogeneity that signifies further research to decode its distinct mutation profile for stratifying patients for targeted therapies [41].

The 5-year DFS and OS rates in the entire cohort were 92.1% and 93.4%, respectively. The favourable prognosis can be attributed to the fact that most of the patients were diagnosed at an early stage with low-grade early-stage disease. These outcomes are widely supported by reported well-established data [2,16,33]. Multivariate Cox regression analysis has shown that advanced FIGO stage and residual disease are prognostic factors independently associated with inferior disease-free and overall survival outcomes. Despite FIGO stage and residual disease, being widely accepted as independent prognosticators, several studies have also reported that tumour grading, BMI, and fertility-sparing surgery represent prognostic factors impacting disease relapse and survival outcomes [29,33,43]. Potential divergent outcomes regarding prognosticators for improved oncologic outcomes can be related to the heterogeneity of the different study populations. It is undeniable that the advent of the molecular classification of endometroid ovarian carcinomas is anticipated to bring new insights in disease prognosis.

Our study did not reveal any significant difference in the DFS and OS, associated with adjuvant chemotherapy. The association between post-operative chemotherapy and improved survival in patients with early-stage low-grade endometrioid ovarian cancer, has been questioned due to the low rate of recurrences observed in patients who have not received adjuvant chemotherapy [15,44]. Based on the outcomes of the parallel randomised trials, ICON -1 and ACTION, adjuvant chemotherapy can improve overall and recurrence-free survival in specific subgroups of early-stage disease. However, subgroup analysis for low/intermediate risk tumours (stage IA grade 1 or 2, IB or IC grade 1) did not provide a clear answer regarding the survival benefits [45,46,47].

Meanwhile, from the results from a retrospective analysis including 4538 patients diagnosed with stage I endometrioid ovarian cancer, administration of chemotherapy was associated with an overall survival benefit for patients with inadequately staged grade 2 tumours [12].

Another population-based study, which examined the correlation between post-operative chemotherapy and overall survival in patients with stage IC grade 1 endometrioid ovarian carcinoma, supports that the 5-year overall survival rate exceeded 90% with statistically non-significant hazard estimates (adjusted hazard ratio 1.54, 95% CI 0.63 to 3.73), in patients who underwent lymph node assessment irrespective of the use of post-operative chemotherapy [48].

A recent retrospective cohort study from the SEER database, including 3957 patients with primary endometrioid OC, has concluded that chemotherapy did not improve the survival time for most patients (*p* > 0.05), except for patients with grade I–II and Stage II–IV disease (overall survival *p* = 0.008, CSS *p* = 0.046). However, subgroup analysis showed that contrary to chemotherapy, lymph node dissection improved prognosis (*p* < 0.05) [49].

In our cohort, lymph node involvement was found in 5.9% of grade 1–2 endometrioid ovarian carcinomas, (3.03%; 1/33 in grade 1 and 8.8%; 3/34 in grade 2 tumours), which is in keeping with the reported data [14,50]. We also found no significance of lymphadenectomy in the overall and recurrence-free survival rates. Due to the low prevalence of nodal metastases in low-grade, early-stage endometrioid ovarian cancer, the value of systematic lymph node dissection represents another issue of controversy.

A retrospective study from Massachusetts General Hospital, which aimed to evaluate the metastatic patterns of primary endometrioid ovarian cancer, concluded that comprehensive lymphadenectomy may not justify the associated morbidity and suggested that the decision to perform lymphadenectomy during primary surgery should depend on clinical factors, including confidence in the strength of the intraoperative pathologic diagnosis, the clinical staging, and the associated risk of potential perioperative morbidity [51]. Several other studies have also reported similar outcomes [43,52].

A recent retrospective study from Bizzarri et al. has shown that staging lymphadenectomy in patients with grade 2 endometrioid ovarian carcinoma was associated with improved DFS and OS. As concluded by the authors, this outcome is supportive of the concept that grade 1 and grade 2 should be considered as two different entities, which could benefit from a different approach in terms of surgical staging [50].

Our study has several limitations, mainly due to the retrospective design, which cannot exclude potential unmeasured bias, and the small sample size, which can explain the wide confidence intervals that suggest a degree of uncertainty in the estimated effects. Therefore, the results should be interpreted with caution as the absence of statistical significance of the different covariates in the recurrence and survival rates might be attributed to the small sample size. In addition, data regarding the molecular profile of the tumours were very limited and, therefore, the low number of events related to the molecular background in the survival analysis has limited our capacity to identify molecular prognostic features in the study population.

## 5. Conclusions

Endometrioid ovarian carcinoma usually affects younger patients with most of the cases representing low-grade carcinoma diagnosed at early stages. The stage at the time of diagnosis and the presence of residual disease following primary surgical management represent independent prognostic factors associated with recurrence or cancer-related death.

Management should aim in maximal cytoreductive effort followed by adjuvant treatment based on the stage of disease, tumour grade, and the completeness of surgical staging. Fertility-sparing options can be considered in stage IA any grade and stage IC1 low-grade tumours and can be acceptable following meticulous counselling in selected cases of IC1 high grade and IC2 tumours irrespective of disease grade.

Larger prospective studies might clarify the precise role and benefits of systematic lymph node dissection and adjuvant chemotherapy in early-stage disease, for optimal oncologic outcomes in patients diagnosed with endometrioid ovarian carcinomas.

In the epoch of molecular profiling, future studies with larger datasets should aim to elucidate further the molecular subtypes, define the prevalence and the prognostic value of major molecular alterations, and develop precise stratification models to plan personalised treatment.

## Figures and Tables

**Table 1:** Clinicopathological characteristics, treatment strategies, and outcomes for patient diagnosed with endometrioid ovarian cancer.

	Descriptives	Value	
		Mean Value/Number	Range/Percentage
Demographic data	1. Age (years)	54	31–86
	<40	8	10.5%
	41–50	24	31.6%
	51–60	21	27.6%
	61–70	12	15.8%
	>70	11	14.5%
	2. BMI	27.4	18–53.3
	Normal 18.5–24.9	34	44.7%
	Overweight 25–29.9	18	23.7%
	Obese ≥30	24	31.6%
	3. Menopausal status		
	premenopausal	20	26.3%
	postmenopausal	40	52.6%
	unknown	16	21.1%
	4. Parity		
	nulliparous	17	22.4%
	parous	59	77.6%
	5. Ethnicity		
	Non-Hispanic white	19	25%
	Hispanic white	17	22.4%
	Black	8	10.5%
	South Asian	11	14.5%
	East Asian	3	3.9%
	Other (including mixed)	5	6.6%
	Not reported	13	17.1%
	6. Comorbidities		
	Hypertension	7	9.2%
	Diabetes	8	10.5%
	COPD/asthma	8	10.5%
	VTE	4	5.3%
	Breast cancer	4	5.3%
	Smoke	13	17.1%
	7. Ca125 status		
	Positive (>35)	38	50%
	Negative (<35)	7	9.2%
	Unknown	31	40.8%
	8. Ca125 category		
	<35	7	9.2%
	35–99	10	13.2%
	100–500	15	19.7%
	>500	4	5.3%
	>1000	9	11.8%
	Unknown	31	40.8%
Histological features	9. Stage (generalised)		
	I	52	68.4%
	II	13	17.1%
	III	11	14.5%
	IV	-	-
	10. Final stage (FIGO 2014)		
	IA	21	27.6%
	IB	2	2.6%
	IC1	8	10.5%
	IC2	15	19.7%
	IC3	6	7.9%
	IIA	7	9.2%
	IIB	6	7.9%
	IIIA1(i)	4	5.3%
	IIIA2	1	1.3%
	IIIB	5	6.6%
	IIIC	1	1.3%
	11. Grade		
	I	33	43.4%
	II	34	44.7%
	III	9	11.8%
	12. Endometriosis		
	Present	41	53.9%
	Non-present	34	44.7%
	Unknown	1	1.4%
	13. Residual disease		
	No residual disease (R0)	72	94.7%
	MRD (<1 cm)	3	3.9%
	GRD (>1 cm)	1	1.4%
	14. Synchronous EEC	15	19.7%
	15. LVSI		
	Present	4	22.2%
	Non-present	14	77.8%
Treatment details	16. Route of surgery		
	Laparoscopy	21	27.6%
	Laparotomy	55	72.4%
	17. Hysterectomy		
	Yes	65	85.5%
	No	11	14.5%
	18. Bilateral salpingo-oophorectomy	69	90.8%
	19. Unilateral salpingo-ophorectomy		
	20. Infracolic omentectomy	67	88.2%
	21. Appendectomy	21	27.6%
	22. Pelvic LN assessment	34	44.7%
	Pelvic LN dissection	3	3.9%
	Pelvic LN sampling	31	40.8%
	23. Para-aortic LN sampling	18	23.7%
	24. Peritonectomy	25	32.9%
	Bladder peritonectomy	14	18.5%
	Pelvic peritonectomy	8	10.5%
	Paracolic peritonectomy	3	3.9%
	25. Other procedures		
	Bowel resection + anastomosis	5	6.6%
	Colostomy	2	2.6%
	Ureteric re-implantation	1	1.3%
	Ureteric stent insertion	1	1.3%
	26. Cytology		
	Positive	14	18.4%
	Negative	62	81.6%
	27. Hospitalisation (days)	4.7 ± 4.1	1–27
	28. Intraoperative complications		
	Haemorrhage	3	3.9%
	Cardiac arrest	1	1.3%
	Respiratory arrest	1	1.3%
	Bladder injury	1	1.3%
	Ureteric injury	1	1.3%
	29. Post-operative complications		
	Pulmonary embolism	1	1.3%
	Respiratory infection	2	2.6%
	Ileus	5	6.6%
	Pelvic collection	2	2.6%
	Wound infection	1	1.3%
	Anaemia	5	6.6%
	Incisional hernia	2	2.6%
	30. Blood transfusion	10	13.2%
	31. Readmission	4	5.3%
	32. Adjuvant chemotherapy		
	Yes	50	65.8%
	No	26	34.2%
	33. Adjuvant chemotherapy type		
	Carboplatin/paclitaxel *	41	53.9%
	Carboplatin only	9	11.8%
	34. Disease-free survival	65.8	3–143
	35. Overall survival	69.1	7–143
	36. Recurrence		
	Yes	13	17.1%
	No	63	82.9%
	37. Death		
	Yes	8	10.5%
	No	68	89.5%

* Two patients received less than six cycles of carboplatin/paclitaxel (one stopped after four cycles due to myelosuppression and one stopped after five cycles due to patient’s choice). BMI: body mass index; COPD: chronic obstructive pulmonary disease; VTE: venous thromboembolism; MRD: minimal residual disease; GRD: gross residual disease; EEC: endometrioid endometrial cancer; LVSI: lymphovascular space invasion: LN: lymph node.

**Table 2:** Characteristics of recurrent cases of endometrioid ovarian carcinoma.

No	Stage	Grade	Synchronous EEC	R0	LN	CT	Site of	DFS	OS	Death
					Assessment		Recurrence			
1	IIIB	3	no	no	yes	yes	Upper abdomen	10	14	yes
							Retroperitoneal LN			
							Liver			
2	IIIB	3	no	no	yes	no *	Pelvis	8	12	yes
							Retroperitoneal LN			
							Rectum			
3	IC3	2	no	yes	no	yes	Pelvic mass	77	88	no
							Retroperitoneal LN			
4	IA	1	Stage IB	yes	no	no	Vaginal vault	50	85	no
			Grade 2				Rectum			
5	IIIA1(i)	3	no	yes	yes	yes	Bladder	17	84	yes
							Rectum			
							Upper abdomen Retroperitoneal LN			
6	IIIA1(i)	1	no	yes	yes	yes	Upper abdomen	11	53	no
							Retroperitoneal LN			
7	IIIA2	3	no	no	yes	no *	Upper abdomen	3	7	yes
							Retroperitoneal LN			
							Rectum			
8	IIIC	3	no	yes	yes	yes	Upper abdomen Retroperitoneal LN	41	66	no
							Liver			
9	IC1	2	no	yes	no	no	Pelvic mass	25	32	no
10	IC2	1	Stage IB	yes	no	yes ^i^	Vaginal vault	38	64	no
			Grade 1				Retroperitoneal LN			
11	IIIB	3	no	no	yes	yes	Upper abdomen	15	27	yes
							Retroperitoneal LN			
12	IC1	1	no	yes	no	no	Pelvic mass	58	68	no
13	IIA	1	Stage IA	yes	no	yes	Pelvis	33	76	no
			Grade 1				Retroperitoneal LN			

EEC: endometrioid endometrial carcinoma, R0: complete resection, LN: lymph node, CT chemotherapy, DFS: disease-free survival (in months), OS: overall survival (in months). * Declined chemotherapy. ^i^ Received vault brachytherapy.

## Data Availability

Data are publicly unavailable due to privacy restrictions.

## Abbreviations

The following abbreviations are used in this manuscript:

OC	Ovarian Cancer
EOvC	Endometrioid ovarian carcinoma
EAOCs	endometriosis-associated ovarian carcinomas
CT	Computerised tomography
MDT	Multidisciplinary team
TCGA	The Cancer Genome Atlas
